# Soil Fertility Map for Food Legumes Production Areas in China

**DOI:** 10.1038/srep26102

**Published:** 2016-05-23

**Authors:** Ling Li, Tao Yang, Robert Redden, Weifeng He, Xuxiao Zong

**Affiliations:** 1 Liaoning Research Institute of Cash Crops, Liaoning Liaoyang, 111000, China; 2 Institute of Crop Science, Chinese Academy of Agricultural Sciences, Beijing, 100081, China; 3 Australian Grains Genebank, Grains Innovation Park, Department of Economic Development, Horsham, Victoria 3401, Australia

## Abstract

Given the limited resources of fossil energy, and the environmental risks of excess fertilizer on crops, it is time to reappraise the potential role of food legume biological nitrogen fixation (BNF) as sources of nitrogen for cropping systems in China. 150 soil samples across 17 provinces and 2 municipalities of China were collected and analyzed. A distribution map of the soil fertilities and their patterns of distribution was constructed. The pH results indicated that soils were neutral to slightly alkaline overall. The soil organic matter (SOM) and the available nitrogen (AN) content were relatively low, while the available phosphorus (AP) and available potassium (AK) contents were from moderate to high. Production areas of food legumes (faba bean, pea, adzuki bean, mung bean and common bean) were clearly separated into 4 soil fertility type clusters. In addition, regions with SOM, AN, AP and AK deficiency, high acidity and high alkalinity were listed as target areas for further soil improvement. The potential was considered for biological nitrogen fixation to substitute for the application of mineral nitrogen fertiliser.

The major food legumes in China, consisting of faba bean (*Vicia faba* L.), pea (*Pisum sativum* L.), common bean (*Phaseolus vulgaris* L.), adzuki bean (*Vigna angularis* (Willd) Ohwi&Ohashi) and mung bean (*Vigna radiata* L.), provide food as dry and fresh grain, fresh pods, as well as fresh and tender stems and leaves. These legumes can play a vital role in biological nitrogen fixation (BNF) and improving soil physical conditions in crop rotation systems. Previous studies mainly focused on breeding and cultivation of food legumes, and ignored their soil environments.

In China, soil quality, fertility and the quantity of arable soil have declined significantly, in part due to long-term use of chemical fertilizers affecting pH (acidic soils are hostile to the majority of legumes) and cation exchange profiles, plus pesticide-related declines in soil-renewing earthworms^1^. For example, from 1997 to 2005, a partial factors analysis of productivity from applied N (PEP_N_) of annual grain decreased from 55 to 20 kg/kg N, which means the N use efficiency has decreased dramatically, and contributed to severe environmental degradation from drainage of excess fertilizer since the 1990s^2^. It is time to change the situation of overdependence on chemical fertilizer and cereal mono-cropping in China. Food legumes will become more important for their BNF nitrogen contribution to intercropping and rotation with cereals and other crops in the future in China^3^.

For optimization of legume nitrogen fixation, adequate levels of available phosphorous and potassium are necessary. The amount of fertilizer applied should at least replace the levels of these minerals removed at grain harvest/hay/grazing, as grain or vegetative components^4^. A low level of external nitrogen supply is needed for legumes during the initial phase of nodulation in the early vegetative phase, before becoming self-sufficient in symbiotically fixed nitrogen^5^. Other key mineral requirements for BNF are availability of phosphorous (P) and potassium (K), where P in particular enhances rhizobial activities of nodulation and nitrogen fixation, as well as increasing grain yield^6^. For nitrogen fixation in faba bean, a range of fertilizer applications of 15–50 kg/ha of P_2_O_5_, and 0.3–0.8 mmlKL^−1^(or mMK) for potassium was recommended^7^. Legumes differ in their growth requirements for P from 0.8–3.0 mM^8^, and also vary in P partitioning between shoot, root and nodules for P use efficiency effects upon BNF^6^. Symbiosis between legumes and *rhizobia* requires P uptake both for plant growth and for nodule establishment and subsequent BNF, with nodules acting as sink for P^9^,^10^, and optimal P stimulating ten-fold accumulation of N in pea^11^.

In this study, we constructed a clear distribution map of soil fertility in food legume production areas of China, and characterized the soil condition of the main production areas for faba bean, pea, adzuki bean, mung bean and common bean, respectively. This work provided the basis for complementary nutrient recommendations in these regional/crop production areas for improvement of the Soil Quality Index (SQI)^12^ through adoption of BNF as a major supplier of nitrogen both direct to legume crops and indirectly to non-legume crops through utilization of legume crop residues.

## Results

### The variation and distribution features of soil nutrient in legume production areas of China

**Figure 1:**
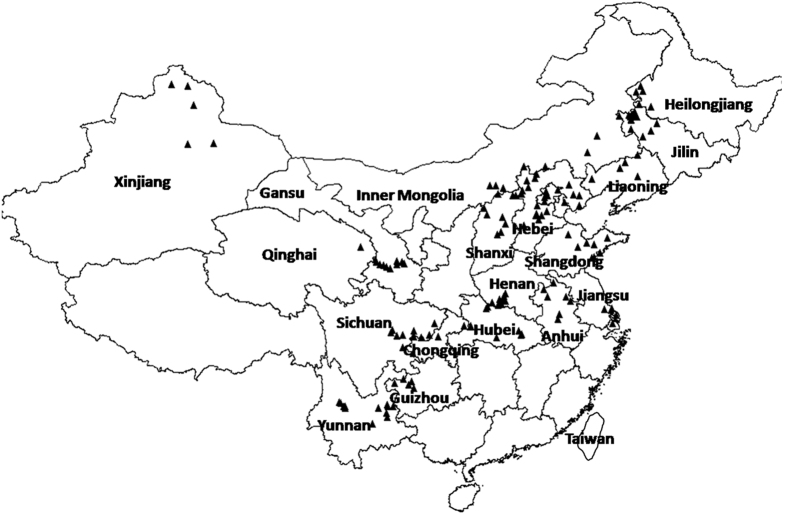
Map of soil samples collecting sites in Greater China created by DIVA_GIS (v7.5) software. Hijmans, R.J., Guarino, L., Mathur P. *DIVA-GIS. Version. 7.5*. http://www.diva-gis.org/ (2012) Data of access: 05/07/2015.

In this study, 150 soil samples from food legumes production areas, were collected across 17 provinces and 2 municipalities of China (Fig. 1) covering nearly all the food legumes cropping regions. It showed that the nutrient contents of soil samples had wide ranges. These were 4.7–9.2 for soil pH, 0.6–7.9% for soil organic matter (SOM), 21–331 mg kg^−1^ for available nitrogen (AN), 1.4–311 mg kg^−1^ for available phosphorous (AP), and 39–487 mg kg^−1^ for available potassium (AK) (Table 1). The minimum coefficient of variation (CV) was 12% for pH in surface soil, the maximum CV was 107% for soil AP content which was far more than other elements. The CVs of soil pH and available nutrients were in order of AP > SOM > AN > AK > soil pH. Perhaps, the high CV for AP was related to the poor mobility of P, which is easily fixed in the soil. In contrast the low CV for K may reflect its high mobility in soil.

Kurtosis is a reflection of the concentration of the sample data. Mathematically it reflected the sharpness (positive kurtosis) versus the flatness (negative kurtosis) of a distribution compared to normal distribution. In this study the distribution of AP had negative kurtosis, whereas for other variables kurtosis was positive. The Soil pH was the closest to a normal distribution.

Skewness reflected the asymmetric degree of the distribution with the mean as the center. The SOM, AN, AP and AK of the soil samples were positively skewed (with a few higher values), whereas soil pH had a negative skew (with a few lower values).

### The distribution feature of soil pH

**Figure 2:**
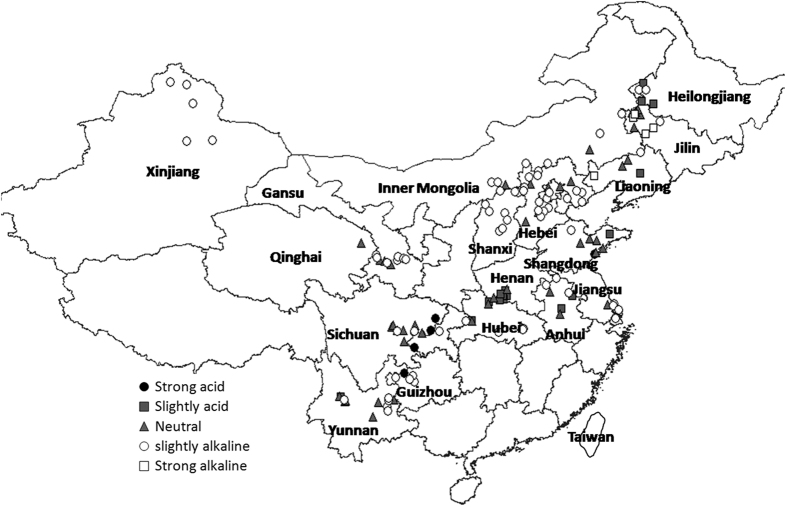
Map of classification of soil pH in food legumes growing areas, Greater China. Created by DIVA_GIS (v7.5) software. Hijmans, R.J., Guarino, L., Mathur P. *DIVA-GIS. Version. 7.5*. http://www.diva-gis.org/ (2012) Data of access: 05/07/2015.

The soil pH of food legumes production areas was neutral overall, suitable for the growth of food legumes. Soil pH had a range 4.7~9.2 and mean 7.4, with 48% of soil samples slightly alkaline, 34% neutral, 11% slightly acid, 4% strongly acidic (from Sichuan and western Guizhou), and 3% strongly alkaline (from Jilin and western Liaoning) (Fig. 2).

### The distribution feature of soil organic matter (SOM)

**Figure 3:**
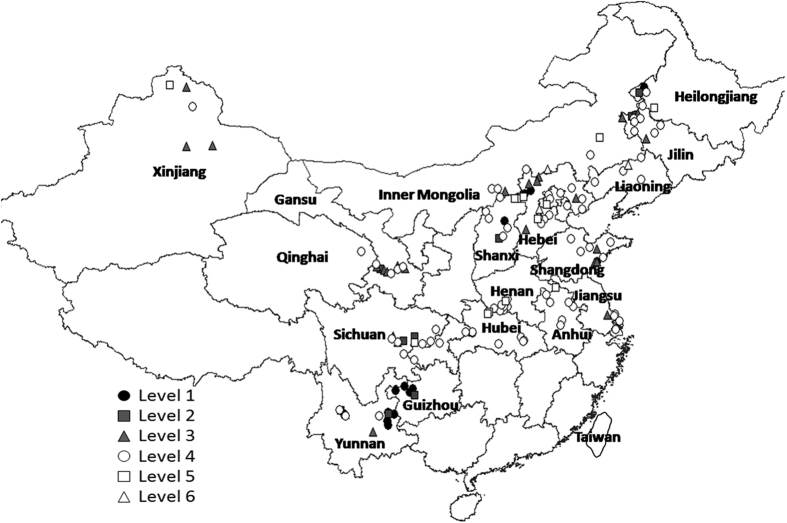
Map of classification of soil organic matter (SOM) in food legumes growing areas, Greater China. Created by DIVA_GIS (v7.5) software. Hijmans, R.J., Guarino, L., Mathur P. *DIVA-GIS. Version. 7.5*. http://www.diva-gis.org/ (2012) Data of access: 05/07/2015.

In this study SOM showed had a range 0.6~7.9%, mean 2.2%, standard deviation (SD)1.4%, and CV 64.6%. Fig. 3 shows the distribution of the SOM levels in soil samples across provinces. These ranged geographically with level 1 (9% with highest SOM) in the border region of Yunnan and Guizhou in mountain valleys and paddy fields, Dingxiang and Datong counties Shanxi, western Heilongjiang, and Huangdao county of Shandong; level 2 (7% with high SOM) in western Jilin and Heilongjiang, Lezhi and Nanchong areas Sichuan, and Zhijin area Guizhou; level 3 (17% with slightly high SOM) widely distributed except in south central China; level 4 (56%) widely distributed; 9% level 5 (9% with low SOM) in Dumeng area Heilongjiang, the adjoining areas of Hebei and Shanxi, Buerjin area of Xinjiang, Gucheng area of Hubei, Xiaoxian area of Anhui and Tongnan area of Chongqing; and level 6 (2% with very low SOM) in central Hebei and northern Liaoning.

### The distribution feature of soil available nitrogen (AN)

**Figure 4:**
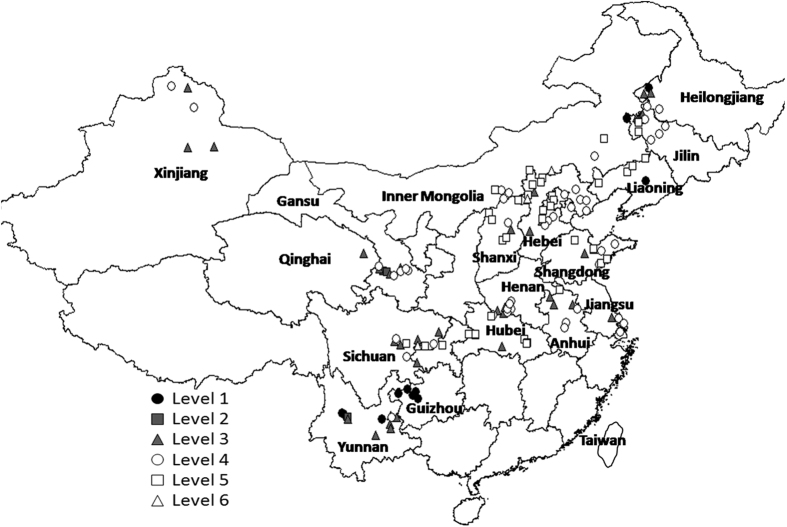
Map of classification of soil available nitrogen (AN) in food legumes growing areas, Greater China. Created by DIVA_GIS (v7.5) software. Hijmans, R.J., Guarino, L., Mathur P. *DIVA-GIS. Version. 7.5*. http://www.diva-gis.org/ (2012) Data of access: 05/07/2015.

AN in food legumes production areas had a range of 21~331 mg kg^−1^, mean 89.9, SD 47.4, and CV 53%. AN was relatively low with level 1 (9% > 150 mg kg^−1^) in Guizhou, Yunnan, Heilongjiang, Liaoning, Jilin, and Inner Mongolia, 2% level 2 (2% with 120~150 mg kg^−1^) in Kangle and Hezheng areas of Gansu, and Xianyun area of Yunnan, levels 3 (28% with 90–120 mg kg^−1^), 4 (33% with 60–90 mg kg^−1^), and 5 (26% with 30–60 mg kg^−1^) each widely distributed, and level 6 (2% < 30 mg kg^−1^, very low AN) in Guyuan area Hebei, Yangyuan area Hebei and Tongnan area Chongqing (Fig. 4). AN was significantly positively related to SOM (r^2^ = 0.304, r = 0.551, P = 0.000).

### The distribution feature of soil available phosphorus (AP)

**Figure 5:**
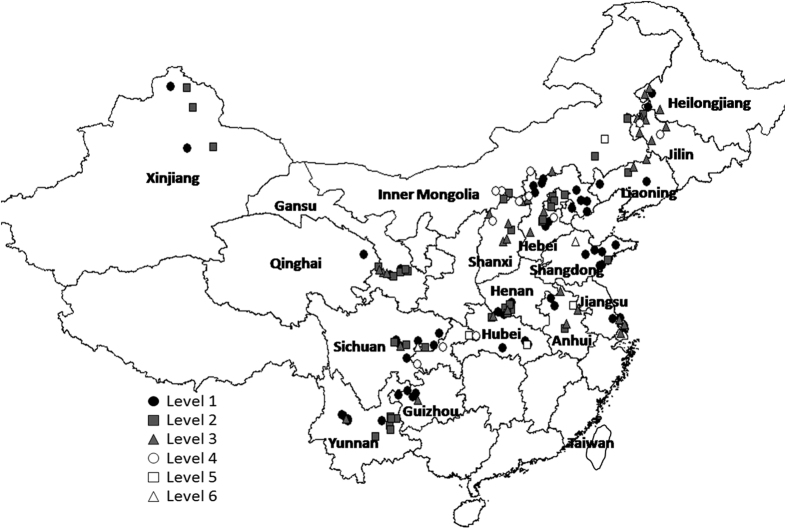
Map of classification of soil available phosphorus (AP) in food legumes growing areas, Greater China. Created by DIVA_GIS (v7.5) software. Hijmans, R.J., Guarino, L., Mathur P. *DIVA-GIS. Version. 7.5*. http://www.diva-gis.org/ (2012) Data of access: 05/07/2015.

AP was relative high, with mean 49.9 mg kg^−1^, range 1.4~311 mg kg^−1^, standard deviation 53, and CV 107%, with level 1 (38% > 40 mg kg^−1^), level 2 (26% with 20~40 mg kg^−1^) and level 3 (24% with 10~20 mg kg^−1^) each widely distributed level 4 (8% with 5~10 mg kg^−1^) in Chongqing, Inner Mongolia, Hebei, Shanxi, and Jilin; level 5 (3% with 3~5 mg kg^−1^) in areas of Wushan Chongqing, Wuhan Hubei, Wuhe Anhui and Chifeng Inner Mongolia (Fig. 5); and level 6 with very low AP value (1% < 3 mg kg^−1^) in Binzhou area of Shandong and Tongnan area of Chongqing.

### The distribution feature of soil available potassium (AK)

**Figure 6:**
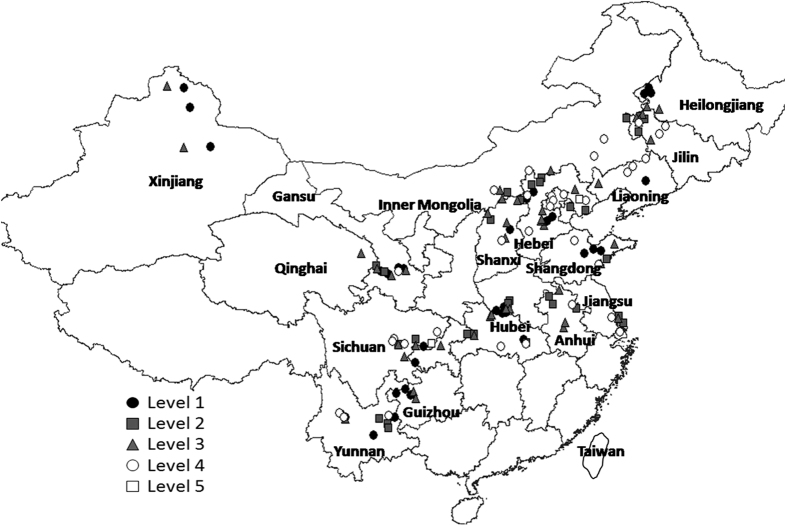
Map of classification of soil available potassium (AK) in food legumes growing areas, Greater China. Created by DIVA_GIS (v7.5) software. Hijmans, R.J., Guarino, L., Mathur P. *DIVA-GIS. Version. 7.5.* http://www.diva-gis.org/ (2012) Data of access: 05/07/2015.

AK content ranged 39~487 mg kg^−1^, mean 157 mg kg^−1^, SD 78, and CV 50%, which was lower than for AN, AP and SOM. Overall, AK was relatively high, level 1 with very high AK (24% > 200 mg kg^−1^), level 2 high AK (21% with 150~200 mg kg^−1^), and level 3 slightly high AK (30% with 100~150 mg kg^−1^), covered nearly all food legumes production areas in China. Level 4 with medium AK (23% with 50~100 mg kg^−1^) were mainly distributed in the North China Plain and northeastern China, and sporadically in south central China. Level 5 with low AK (2% with 30~50 mg kg^−1^), occurred in Qianxi area Hebei and Dazhu area of Sichuan. No legumes production area had very low AK (level 6 with  <30 mg kg^−1^) (Fig. 6).

### Characterization of the faba bean production areas in China

**Figure 7:**
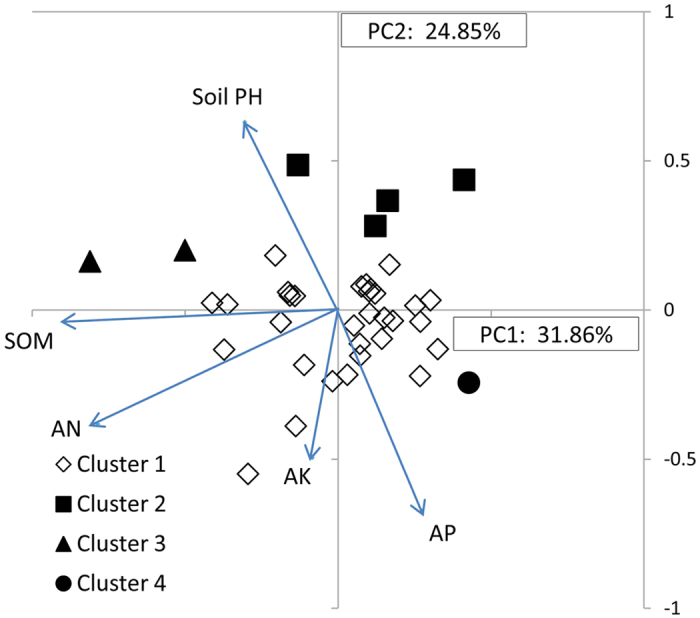
Principal components analysis of 38 soil samples of the faba bean production areas. Variable factor loadings for PC1 and PC2 were soil pH and soil nutrient variables and presented as vectors.

Principal components analysis (PCA) of soil nutrients in faba bean production areas explained 57% of the variance in the first two components. PC1 was dominated by SOM and AN accounting for 32% of variance, while AP and soil pH were important variables in PC2 which accounted for 25% of variance (Fig. 7). Cluster analysis showed that 38 soil samples from the faba bean production areas covering 11 provinces were classified into 4 clusters (Fig. 7 and Table 2), merging at a similarity level of 84% (Fig. S1), and differing in soil pH, SOM, AN, AP and AK. Cluster 1 with a high SQI of 1.20 (SD 0.17) and relatively high mineral levels (Table 2 and Table S1) comprising 82% of soil samples, was mostly in Yunnan, Sichuan, Hubei, Qinghai and Gansu. Cluster 1 was located around the origin of the PCA bi-plot (Fig. 7) and also included low pH, high P&K sites. Cluster 2, with 4 soil samples from Anhui, Jiangsu, Hebei and Chongqing respectively, had the lowest SQI, moderately alkaline pH, low SOM, AN, AP, and high AK, and mapped to the upper section of PC2. Cluster 3, comprising 2 soil samples from Zhanyi area and Dali area of Yunnan province, with a slightly alkaline pH, high SOM and AN, moderate AP and low AK levels, had the highest SQI, and mapped along the extreme negative axis of PC1 in Fig. 7. The SQI of cluster 4 was similar to cluster 2 for SQI, but significantly lower than clusters 1 and 3. One cluster 4 soil sample from Dazhu area of Sichuan, with very low pH, low SOM, AN and AK, and high AP, the most acidic environment, was in the lower right quadrant of Fig. 7.

### Characterization of the pea production areas in China

**Figure 8:**
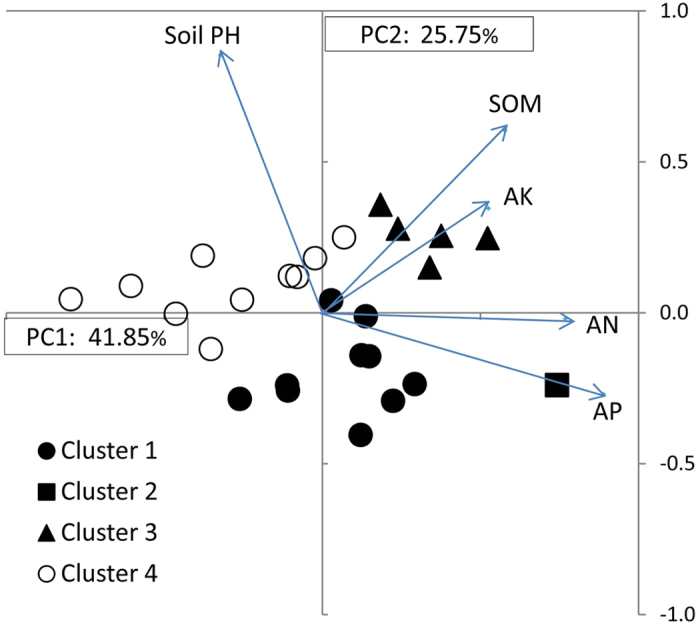
Principal components analysis of 26 soil samples of the pea production areas. Variable factor loadings for PC1 and PC2 are soil pH and soil nutrient variables and presented as vectors.

The first two components of the PCA explained 77% of the variance. The most important characters in the first component were AP and AN, and in the second component pH and SOM. Cluster analysis of 26 soil samples was found to separate pea production areas over 10 provinces and 1 municipality (Fig. 8) into 4 clusters, merging at the similarity level (86%) (Fig. S2). Cluster 1 (10 samples) with low SQI (1.18), high AP, neutral pH, below average SOM, and above average AN and AK, plotted to the lower-central portion of Fig. 8. Cluster 2, with the combination of the highest AN, AP and AK, slightly high SOM and slightly acid pH (Table 3), had 1 site, Dengta area in Liaoning, and was located furthest along the positive axis of PC1. It had the highest SQI indicating better soil quality. Cluster 3, (5 samples) with high SQI, had high levels of AK and SOM, moderate levels of AN and AP, and slightly alkaline pH conducive to BNF, occurred in Jiangchuan area of Yunnan, Anding and Lintao areas of Gansu, Dingxiang area of Shanxi and Mulei area of Xinjiang, and mapped in the upper right quadrant of Fig. 8. Cluster 4 (10 soil samples) with low mineral and SOM content, had the significantly lowest SQI, was located in the central to eastern provinces, such as Chongqing, Hubei, Anhui, Jiangsu, Shandong and southern Gansu (Fig. 8). These were plotted along the negative axis to the origin of PC2.

### Characterization of the adzuki bean production areas in China

**Figure 9:**
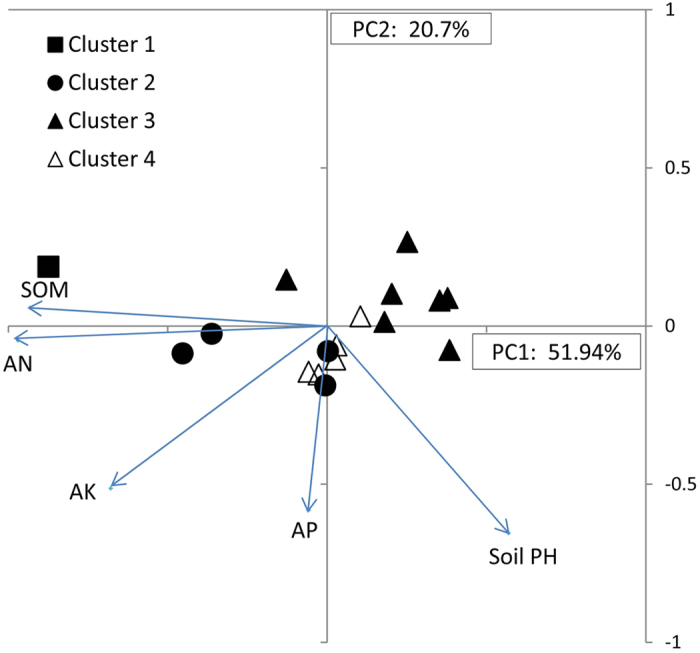
Principal components analysis of 17 soil samples of the adzuki bean production areas. Variable factor loadings for PC1 and PC2 are soil pH and soil nutrient variables and presented as vectors.

The first two components of the PCA explained 73% of the variance. The most important traits in the first component were SOM and AN, in the second component AK and pH. Cluster analysis of 17 soil samples was found to separate adzuki bean production areas across 3 provinces and 1 municipality (Fig. 9) into 4 clusters, merging at the similarity level of 86% (Fig. S3). Clusters 3 and 4 with low SQI had relatively low fertility and slightly alkaline soils, mostly in Hebei, Beijing, Liaoning and Inner Mongolia, comprising 71% of the adzuki bean production areas. These were located around the origin of the PCA bi-plot and extent to the upper right quadrant of Fig. 9. Cluster 2 with high SQI, was mainly from Hebei and Heilongjiang, with the highest AK, high AP, and above average AN and SOM (Table 4), and mapped to the lower left quadrant of Fig. 9. Cluster 1 in Gannan area of Heilongjiang, had the highest SQI representing the highest fertility soil, mapped furthest along the negative axis of PC1 of Fig. 9.

### Characterization of mung bean production areas in China

**Figure 10:**
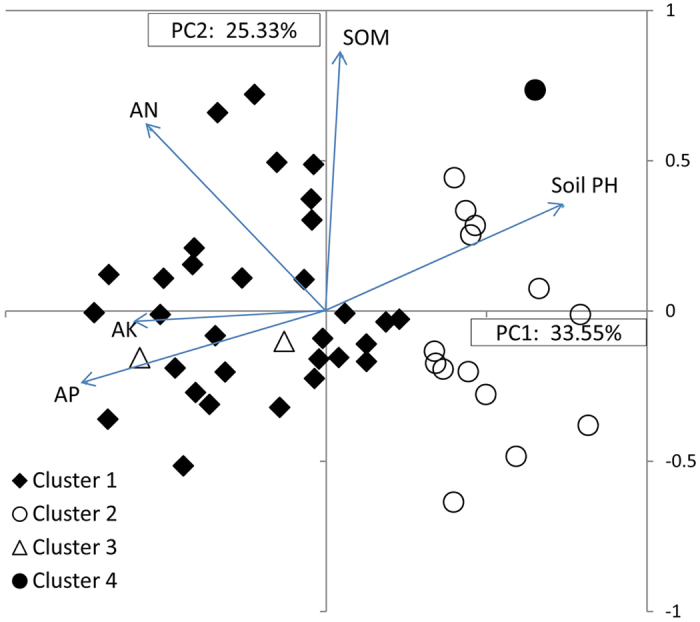
Principal components analysis of 48 soil samples of the mung bean production areas. Variable factor loadings for PC1 and PC2 are soil pH and soil nutrient variables and presented as vectors.

Principal components analysis showed that 59% of variance was explained by the first two components of the PCA. PC1 was dominated by AP, PC2 was dominated by SOM and AN. Mung bean production areas with soil samples from 48 sites across 10 provinces and 1 municipality classified into 4 clusters (Fig. 10), merging at the similarity level of 86% (Fig. S4). Cluster 1 with relatively high AN, AP and AK, neutral to acidic pH range and below average SOM plotted to the negative side of PC1 and extended a little to the positive side of PC1 (Fig. 10). SQI values of different clusters had a range of 0.80~1.05, significant difference existed only between cluster 1 and cluster 2. Cluster 1 (SQI 1.1) included more than half of the mung bean production areas in the central to northern provinces including Henan, Anhui, Shandong, Hebei, Shanxi, Liaoning, Jilin, Inner Mongolia and Heilongjiang. Cluster 2 with the lowest SQI was located on the positive side of PC1, and geographically overlapped cluster 1 in Hubei, Hebei, Beijing, Liaoning, Jilin and Inner Mongolia. Cluster 2 also had the lowest SOM (Table S1), below average AN, above average AP and AK, and slightly alkaline soils. Cluster 3 with neutral-low pH, moderate to high AP and moderate AN, AK and SOM (Table 5), was mapped in the lower left quadrant of Fig. 10. Cluster 4 with only 1 soil sample from Datong in Shanxi with the highest SOM (7.8%), lowest AP, low AN and AK and slightly alkaline soil, was located in the upper right quadrant of Fig. 10.

### Characterization of the common bean production areas

**Figure 11:**
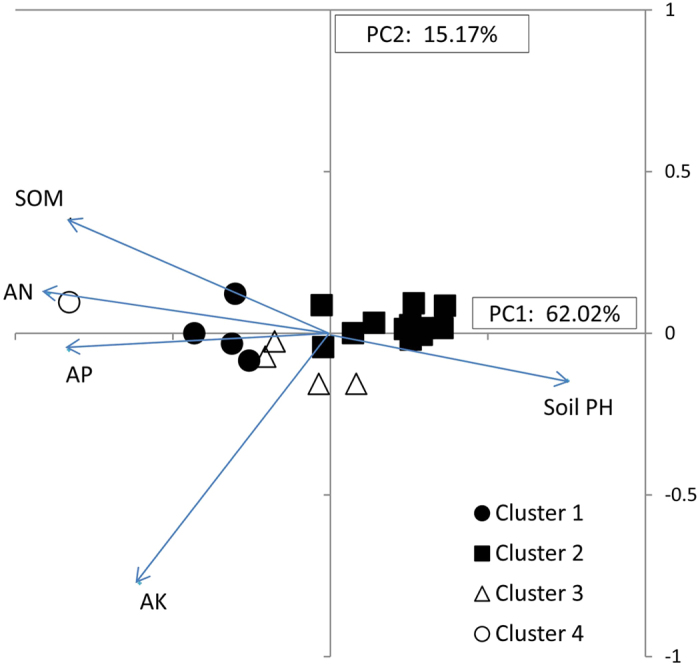
Principal components analysis of 21 soil samples of the common bean production areas. Variable factor loadings for PC1 and PC2 are soil pH and soil nutrient variables and presented as vectors.

The first two components of the PCA explained 77% of the variance. The most important characters were SOM, AN and AP in the first component, and AK in the second component. The common bean production areas with 21 soil samples were distributed in 7 provinces and separated into 4 clusters (Fig. 11), merging at the 86% similarity level (Fig. S5). The SQI values were higher than for mung bean. Cluster 1 with high mineral levels, high SOM and slightly alkaline pH suitable for growing common bean, had the highest SQI (Table 6), plotted to the left of PC2. Cluster 1sites with low SQI were distributed in Yunnan and Guizhou (Table S1). Clusters 2 and 3 with below average SOM and AN, moderate to high AP and AK, and neutral pH, were in the Zhijin area of Guizhou and in northern China (Xinjiang, Shanxi, Hebei, Shandong and Inner Mongolia), comprising 76% of the common bean production areas. Clusters 2 and 3 were mapped around the origin of the PCA. Cluster 4 (1 site) with the highest SOM, AN, AP, high AK, and strongly acidic pH, occurred in the Hezhang area of Guizhou (Cluster 4), which mapped furthest along the negative axis of PC1.

### Discussion and conclusion

#### The overview of Chinese main production areas for food legumes

The soils map showed that the food legumes production areas were mostly neutral to slightly alkaline, very suitable for growing legumes and favourable for rhizobial nitrogen fixation, and AN and SOM contents were generally low. Though legume crops have nitrogen fixation capacity, the species and quantity of soil microorganisms has decreased dramatically. Rhizobial nitrogen fixation has been inhibited by an increased use of chemical fertilizers, which have displaced organic fertilizer to result in environmental degradation and soil pollution^13^,^14^.

Soil pH is one of the most important parameters. It plays a vital role in the availability of nutrients to plant roots, nutrient run-off, leaching and microbial efficiency (http://extension.umd.edu/hgic/soils/soil-testing). All crops show different pH preferences. The pH of the soil which directly affects soil properties and availability of nutrients, is easily determined (http://www.esf.edu/pubprog/brochure/soilph/soilph.htm). Soils with high acidity tend to have toxic amounts of aluminum and manganese and to be hostile to most legume crops. Plants need calcium and moderate alkalinity, but most minerals are more soluble in acid soils. Soil organisms are hindered by high acidity, and most agricultural crops do best with mineral soils of pH 6.5 or with organic soils of pH 5.5^15^. The mostly neutral to slightly alkaline pHs were, very suitable for the growth of food legumes. Strongly acidic sites were Hezhang Guizhou, Dazhu and Dazhou in Sichuan, Yongchuan Chongqing, Sheqiin Henan, and Huangdao Shandong. Strongly alkaline sites were Taonan, Tongyu, Changling and Zhenlai in Jilin, and Kazuo Liaoning. These areas should be considered for a long term remediation project, with yearly measurement of soil pH and gradual adjustments Such as the addition of lime or hardwood ash help to raise the soil pH. For alkaline soils the addition of sulphur powder or ferrous sulfate help to lower the soil pH.

It is now widely recognized that SOM plays an important role in soil biology (provision of substrate and nutrients for microbes), chemical (buffering and pH changes) and physical (stabilization of soil structure) properties^16^. In fact, these properties, along with soil organic carbon (SOC), N and P, are considered critical indicators for the health quality and purpose of the soil (Karlen *et al*.^17^, Norfleet *et al*.^18^). Reeves^19^ showed that soil organic carbon is the most often reported attribute from long-term agricultural studies and is chosen as the most important indicator of soil quality and agronomic sustainability because of its impact on other physical, chemical and biological indicators of soil quality. In this study, the sites with very high organic matter were in Yunnan, Guizhou and a few areas of northeastern China. In Yunnan, faba bean is the main cultivated legume, sown in rotation after rice with notillage into the remaining stubble after removing grain. Other studies in northern China, suggest that long-term no-tillage with straw cover significantly improved both top soil conditions and whole of soil profile SOM, and this improvement was obvious in different layers^20^. The sites with very low soil organic matter were in arid areas, Zhangwu Liaoning, and central Hebei, where mung bean and adzuki bean are planted. These areas would benefit from increased application of organic fertilizer.

Nitrogen (N) is the most critical element obtained by plants from the soil and is a bottleneck in plant growth. N can promote crop root growth, absorption and utilization of soil nutrients. Available nitrogen (AN) content and crop growth are closely related. AN content can reflect the instantaneous release of soil N, and provides a guide to fertilizer application^21^. In this study, the distribution of the sites with very high available nitrogen is the same as for SOM, in Yunnan, Guizhou and a few areas of northeastern China. The sites with very low AN were in Tongnan Chongqing, central Hebei. The results showed the distribution trends of AN and SOM were similar and correlated (r^2^ = 0.304, r = 0.551, P = 0.000). Zhou^22^ also found there was a highly significant association between soil available nitrogen and organic matter in the maize zone of Jilin Province, and similarly for the tobacco growing soils in Yunnan (Wang^23^). Hallett *et al*.^24^ demonstrated that mineral N fertilizers promote a rapid turnover of the light carbon fraction and growth of fungi and hyphae. The distribution of organic C and N was affected by many factors including crop rotation^25^, type and length of tillage^26^,^27^,^28^,^29^, and fertilizer applications^30^. Therefore management measures should be considered which can raise SOM content and enhance nitrogen fixation through utilization of legumes.

Phosphorus is the second most critical plant nutrient. It is generally unavailable in the form of phosphates of low solubility. Total phosphorus is about 0.1 percent by weight of the soil, but only one percent of that is available AP which could be directly absorbed by crops, the critical factor to determine the utilization of phosphate fertilizer^31^. Soil available phosphorus content was mostly high, but with larger variation, since phosphorus with poor mobility may be easily fixed in the soil. The sites with low available phosphorus were Tongnan Chongqing and Binzhou Shandong with pea cultivation. In order to promote the growth of mycorrhiza and increase the utilization ratio of phosphate fertilizer, farmers of these areas are advised to increase not only phosphate fertilizer but also organic fertilizer and to inoculate with *rhizobia*. Because of very low diffusion coefficient (1 × 10^−10^~1 × 10^−8^) and poor mobility of phosphorus in the soil, plants can absorb available phosphorus of from the soil rhizosphere within 1~4 mm from the root. However, in clay soils with strong adsorption only the rhizosphere soil within 1 mm from the root can provide phosphorous^32^.

K as an essential nutritional element for plant growth, participates in nearly all the physiological and biochemical processes of crops, increasing the strength of stem tissues, improving tolerance to biotic and abiotic stresses, and enhancing the ability to fix nitrogen^33^. Soil AK content is one of the main indices to access the capacity of the soil to provide K. Soil available potassium content was mostly high, CV was lower than for organic matter and other nutrients which may be related to high mobility of the K ion. The sites with lower potassium were Dazhu Sichuan with faba bean cultivation, and Qianxi Hebei with adzuki bean cultivation. All plant uptake of potassium is obtained directly from the soil by roots, and farmers can increase potassium application for improved yield where soils are low in AK.

#### The distribution style of food legume types

**Figure 12:**
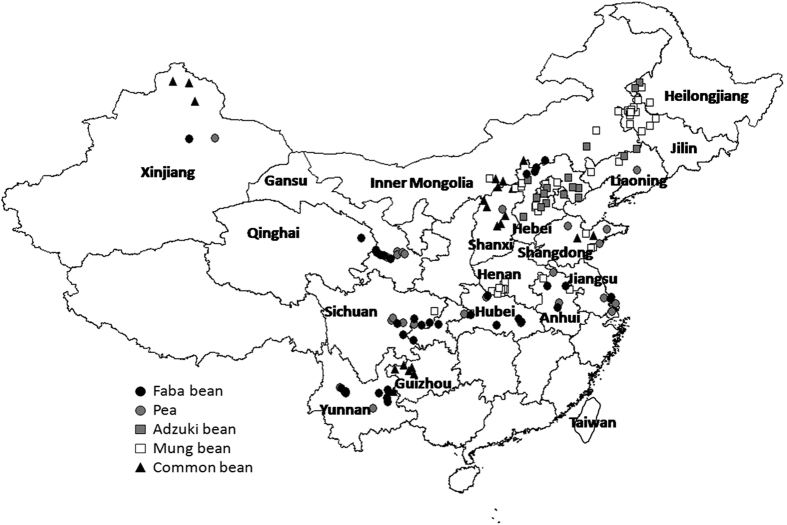
The distribution map of 150 production areas of five different food legumes, Greater China. Including 38 faba bean sites, 26 pea sites, 17 adzuki bean sites, 48 mung bean sites and 21 common bean sites. The map was created by DIVA_GIS (v7.5) software. Hijmans, R.J., Guarino, L., Mathur P. *DIVA-GIS. Version. 7.5.* http://www.diva-gis.org/ (2012) Data of access: 05/07/2015.

The aim of this study was to obtain the distribution features of soil fertility for improved soil management in pea, faba bean, adzuki bean, mung bean and common bean production areas (Fig. 12 and Table 7).

The sowing areas of faba bean and pea for vegetable and grain which accounted for 43% of food legumes in China, were distributed widely. 80% of faba beans and peas were grown for vegetable production on high fertility soils, and additional nitrogen was applied in batches after every harvest, to result in excessive fertilization. However, 20% faba bean and pea grown for dry seed production were planted in intermediate to poor fertility soils such as in Xinjiang, Gansu and Shanxi, with only a reduced or no basal application of compound fertilizer, nor any in-crop application of nitrogen fertiliser. However, there was no obviously difference in SOM, AN and AP between vegetable and grain areas (Table 8). There were differences amongst the clusters of soil types for faba bean and pea production, and these need to be further analysed for possible differences between grain and vegetable production, as well as the availability of soil moisture and irrigation.

Adzuki bean and mung bean, for grain production only, were mainly planted as cash and relief crops in low fertility soils of northeastern and northern China, accounting for 43% of food legume areas in China. Traditionally little or no basal (nor in-crop) fertilizer was applied.

Common bean, mainly used as grain, only accounted for 14% of food legumes production areas in China. Production management with extensive management and low fertilizer application was similar to adzuki bean and mung bean.

This study showed that the SQI had different means and ranges between legumes. These were respectively 1.2 (0.6–1.6) for faba bean, 1.1 (0.4–1.6) for pea, 1.0 (0.6–1.7) for adzuki bean, 1.0 (0.5–1.3) for mung bean, and 1.6 (0.8–2.4) for common bean. The SQI of production areas for the five legumes were in order of common bean > faba bean > pea > adzuki bean > mung bean.

There was little attention in all legume production to capturing the benefits of biological nitrogen fixation (BNF) from symbiotic *rhizobia* inoculation of roots to supply the crop nitrogen. Experiments will be needed to compare the yields and economics of optimal management for BNF in contrast to applied nitrogen fertilizer, as well as the respective environmental risks.

The extensive production of dry seed for legumes may already be dependent on BNF from indigenous *rhizobia*. But with little or no basal fertilizer application BNF could be enhanced by ensuring that the complementary levels of phosphorous (P) and potassium (K) nutrients are adequate. Hence a basal application of P & K fertilizer, plus starter levels of N to assist seedlings until rhizobial symbiosis is established, would facilitate the availability of NPK nutrients to legume crops provided that there was sufficient indigenous *rhizobia.*

#### Supply of crop nitrogen with BNF and of complementary fertilisers

Legume crops differ in BNF potential in the field, which is greatest for soy bean followed by faba bean, pea, chickpea, lentil and common bean^34^. The legume BNF system provides an important opportunity to reduce the input of nitrogen fertilizer and associated costs, to achieve improved efficiency in fertilizer use in both dryland and irrigated agriculture. This also improves AN for rotation crops such as wheat, enabling a reduction in fertilizer input, a double benefit of legume BNF. An important pre-condition is adequate AP and AK which can be adjusted with fertilizer input according to soil analyses.

In Brazil little or no nitrogen fertilizer is applied on 23 million ha of soybeans, which are successfully reliant on both applied *rhizobia* inoculum and other nitrogen fixing organisms (PGRB) such as *Azospirilla*, for 80% of the crop nitrogen requirements up to 300 Kg/ha, with very large savings in crop input costs^34^. BNF benefits for grain yield have been demonstrated in Vietnam with rhizobial inoculation across a variety of legume crops^35^. Rotation benefits for following wheat crops have been shown in Australia from BNF in chickpea and in faba bean crops^36^. These ranged from 10–40% increase in wheat grain yield with up to 40 kg/ha of additional nitrogen available from BNF.

The levels of indigenous *rhizobia* in the soils of China can be built up by initial cultivation of legumes in the absence of nitrogen fertilizer, to provide satisfactory levels of *rhizobia* inoculums for subsequent legume crops^37^,^38^,^39^. An initial survey of unfertilized/low nitrogen input legume crops at the flowering would indicate the extent of root nodulation by indigenous *rhizobia*, and assist in BNF planning for the future. Due consideration could be given in the future to commercial production of *rhizobia* and its distribution in legume production regions, in conjunction with adequacy of AP and AK, to optimize benefits from BNF for farming systems in China.

## Materials and Methods

### Soil samples collection and measurement

150 soil samples from food legumes production areas, were collected across 17 provinces and 2 municipalities of China. These enabled a soil fertility map to be constructed for the food legume production areas in China. A representative random sampling-method^40^ was used for all soil samples, which were collected from 0–20 cm depths after harvest of food legumes. Each soil sample was comprised of a mixture of five cores taken randomly from within a 20 m^2^ plot. After air-drying, soil samples were sieved (0.25 mm openings) into bags which were sent to Liaoning Institute of Cash Crops for analyses of soil pH, soil organic matter (SOM), available nitrogen (AN), available phosphorus (AP) and available potassium (AK).

The potassium dichromate-volumetric method was used to determine SOM^41^. AN was measured using alkaline hydrolysismethod^42^. AP (P Olsen) was extracted by shaking 1.5 g of soil for 30 min at 20 °C in 100 ml of a 42% NaHCO_3_ solution pH 8.5 and determined by theo-Sb colorimetric method according to Olsen^43^. AK was extracted with NH_4_OAc and determined using a flame photometer^44^. Soil pH was measured in 1:2.5 W/W extractions using 0.01 M calcium chloride solution with the methods described by Van Reeuwijk (2002)^33^.

### The classification standard of soil nutrient and soil pH

Soil pH was graded including 5 response levels, strongly acid with PH value <5.5, slightly acid with a range of 5.5~6.5, neutral with a range of 6.5~7.5, slightly alkaline with a range of 7.5~8.5, and strongly alkaline with PH value > 8.5 respectively (Table 9), using the standard method^45^ according to soil nutrient classification standard of the second national soil survey in China^46^.

The soil nutrients were classified from level 1 to level 6:

SOM, level 1 - extremely high (>4%), level 2 - very high (3~4%), level 3 - high (2~3%), level 4 - medium (1~2%), level 5 - low (0.6~1%), level 6 - very low (<0.6%);

AN, level 1 with extremely high (>150 mg kg^−1^), level 2 - very high (120~150 mg kg^−1^), level 3 - high (90~120 mg kg^−1^), level 4 - medium (60~90 mg kg^−1^), level 5 - low (30~60 mg kg^−1^), level 6 - very low (<30 mg kg^−1^);

AP, level 1 - extremely high (>40 mg kg^−1^), level 2 - very high (20~40 mg kg^−1^), level 3 - high (10~20 mg kg^−1^), level 4 - medium (5~10 mg kg^−1^), level 5 - low (3~5 mg kg^−1^), level 6 - very low (<3 mg kg^−1^);

AK, level 1 - extremely high (>200 mg kg^−1^), level 2 - very high (150~200 mg kg^−1^), level 3 - high (100~150 mg kg^−1^), level 4 - medium (50~100 mg kg^−1^), level 5 - low (30~50 mg kg^−1^), level 6 - very low (<30 mg kg^−1^) (Table 10).

### Statistical analysis

The data were analyzed using Genstat^47^ (Genstatv13). Following test of normality distribution, some variables were log or square root transformed to improve normality. Principal components analysis (PCA) and cluster analysis (CA) were used to classify and group the soil types of different food legumes production areas in China. The cluster analysis was conducted using the Euclidean distance matrix to form the clusters. Average linkage method was used as a cluster joining criterion in the dendrogram^48^. PCA based on the correlation matrix was used to construct a bi-plot of soil samples (PC scores) and soil nutrients (PC factor loadings, shown as bi-plot vectors)^32^,^33^,^34^.

At the same time, Soil Quality Index (SQI) was estimated using principal component analysis^12^,^49^,^50^. In this study, we included all the five indicators to create a minimum data set (MDS). We chose first three PCS with high eigenvalues to define the ‘highly weighted’ variables as the highest weighted variable under a certain PC. After selection of parameters for the MDS, all selected observations were transformed into numerical scores (ranged 0–1) by employing linear scoring functions^49^. Soil parameters were divided into groups based on two mathematical algorithm functions: (a) ‘more is better’ (e.g., SOM, AN, AP and AK) and (b) ‘optimum’ (e.g., PH)^12^. A weighted additive approach was used to integrate them into indices for each soil^49^,^50^. Each PC explained a certain amount of variation in the dataset, which was divided by the maximumtotal variation of the all PCs selected for the MDS to get a certain weightage value under a particular PC^49^. The SQI was calculated using weighting factors for each scored MDS variable according to the following formula:


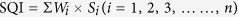


Where *W* is the PC weighting factor and *S* is the indicator score. We compared the calculated SQI means of different groups using Student’s *t* for each legume respectively. We assumed that higher index scores meant better soil quality or greater performance of soil functions.

The soil samples collection sites and the distribution map of soil nutrients were drawn with DIVA_GIS (v7.5)^51^. County level data for China including administrative boundaries were downloaded from http://www.diva-gis.org/Data. The statistical feature values of soil nutrients were calculated by Excel (2007).

## Additional Information

**How to cite this article**: Li, L. *et al*. Soil Fertility Map for Food Legumes Production Areas in China. *Sci. Rep.* **6**, 26102; doi: 10.1038/srep26102 (2016).

## Supplementary Material

Supplementary Information

## Figures and Tables

**Table 1:** The statistical feature values of soil nutrients.

Soil sampling	Mean	Mid	Mode	Min	Max	Stdev	CV(%)	Kurtosis	Skew
pH	7.4	7.6	7.8	4.7	9.2	0.9	11.6	1.02	−1.0
SOM (%)	2.2	1.7	1.9	0.6	7.9	1.4	64.6	4.9	2.1
AN (mg kg^−1^)	89.9	81.5	42.0	21.0	331.0	47.4	52.8	6.5	2.2
AP (mg kg^−1^)	49.9	28.1	–	1.4	310.5	53.3	106.8	−5.5	2.1
AK (mg kg^−1^)	156.6	139.7	139.7	38.5	486.7	77.9	49.8	2.6	1.3

**Table 2:** Soil characterization of different groups for faba bean production area in China.

Cluster	Number of sites	Soil pH	SOM(%)	AN	AP	AK	SQI
				(mg kg^−1^)	(mg kg^−1^)	(mg kg^−1^)	
1	31	7.27 ± 0.65bA	2.26 ± 1.04bB	97.17 ± 43.00bB	56.42 ± 37.22bAB	169.59 ± 50.9aA	1.20 ± 0.71aA
2	4	8.08 ± 0.22aA	1.18 ± 0.36cB	64.50 ± 37.08bB	10.24 ± 5.78cB	111.50 ± 17.71bAB	0.80 ± 0.14bB
3	2	7.85 ± 0.07abA	5.94 ± 0.28aA	216.25 ± 162.28aA	17.95 ± 7.47bcB	68.14 ± 10.95cBC	1.34 ± 0.07aA
4	1	4.9cB	1.33bcB	71.66abAB	112.85aA	38.55cC	0.90 ± 0.00bAB

The different capital and lowercase letters mean significantly different at 0.01 and 0.05 probability levels, respectively.

**Table 3:** Soil characterization of different groups for pea production area in China.

Cluster	Number of sites	Soil pH	SOM(%)	AN(mg kg^−1^)	AP(mg kg^−1^)	AK(mg kg^−1^)	SQI
1	10	6.79 ± 0.44bB	1.73 ± 0.65bB	91.83 ± 62.02abcABC	70.65 ± 78.02bB	113.69 ± 42.67bB	1.18 ± 0.16bB
2	1	6.20bB	2.04abAB	193.00aA	310.47aA	246.77aA	1.55 ± 0.00aA
3	5	7.9 ± 0.46aA	3.0 ± 0.84aA	99.5 ± 14.36bB	70.4 ± 66.27bB	246.6 ± 71.81aA	1.35 ± 0.12abAB
4	10	8.04 ± 0.22aA	1.58 ± 0.49bB	60.67 ± 20.60cC	14.06 ± 11.84cB	122.15 ± 41.43bB	0.84 ± 0.25cC

The different capital and lowercase letters mean significantly different at 0.01 and 0.05 probability levels, respectively.

**Table 4:** Soil characterization of different groups for adzuki bean production area in China.

Cluster	Number of sites	Soil pH	SOM(%)	AN	AP	AK	SQI
				(mg kg^−1^)	(mg kg^−1^)	(mg kg^−1^)	
1	1	6.0bB	5.4aA	215.0aA	19.6bB	244.5aA	1.66 ± 0.00aA
2	4	7.98 ± 0.44aA	2.91 ± 1.61abAB	97.78 ± 19.57bB	31.06 ± 22.62bB	242.36 ± 13.82aA	1.23 ± 0.28abAB
3	7	7.6 ± 0.62aA	1.1 ± 0.50bcB	62.4 ± 19.47cB	19.1 ± 6.98bB	78.7 ± 21.13bB	0.80 ± 0.19cC
4	5	7.58 ± 0.04aA	1.65 ± 0.43bB	77.95 ± 4.20bcB	120.25 ± 11.03aA	127.07 ± 67.21bA	1.00 ± 0.04bB

The different capital and lowercase letters mean significantly different at 0.01 and 0.05 probability levels, respectively.

**Table 5:** Soil characterization of different groups for mung bean production area in China.

Cluster	Number of sites	Soil pH	SOM (%)	AN	AP	AK	SQI
				(mg kg^−1^)	(mg kg^−1^)	(mg kg^−1^)	
1	31	6.90 ± 0.89bB	1.79 ± 0.90bB	92.65 ± 32.84aA	53.33 ± 49.18aA	169.67 ± 49.27aA	1.05 ± 0.14aA
2	14	7.92 ± 0.78aA	1.43 ± 0.47bB	59.73 ± 21.59bB	15.52 ± 9.41bB	102.01 ± 54.03bB	0.80 ± 0.15bB
3	2	4.9 ± 0.14cC	2.0 ± 0.24bB	81.9 ± 14.48abAB	111.8 ± 98.54aA	69.9 ± 13.63bB	1.00 ± 0.00abAB
4	1	7.80aA	7.77aA	51.19aA	6.37abAB	84.32bAB	0.86 ± 0.00abAB

The different capital and lowercase letters mean significantly different at 0.01 and 0.05 probability levels, respectively.

**Table 6:** Soil characterization of different groups for common bean production area in China

Cluster	Number of sites	Soil pH	SOM(%)	AN	AP	AK	SQI
				(mg kg^−1^)	(mg kg^−1^)	(mg kg^−1^)	
1	4	7.6 ± 0.54aA	6.17 ± 1.57bB	168.75 ± 48.86aAB	72.66 ± 44.05bA	308.98 ± 128.15aA	2.26 ± 0.19aA
2	12	7.9 ± 0.28aA	1.78 ± 0.92cC	72.06 ± 33.30bC	18.68 ± 13.43cB	132.76 ± 41.83bB	1.23 ± 0.34bB
3	4	7.6 ± 0.46aA	1.80 ± 0.34cC	85.30 ± 10.27bB	89.60 ± 70.14abA	346.00 ± 127.00aA	1.58 ± 0.15bB
4	1	4.9bB	7.71aA	254.00aA	186.51aA	244.53aA	2.35 ± 0.00aA

The different capital and lowercase letters mean significantly different at 0.01 and 0.05 probability levels, respectively.

**Table 7:** The soil nutrients content of sites for production of different food legumes.

Crop	Number of sites	Soil pH	SOM(%)	AN (mg kg^−1^)	AP (mg kg^−1^)	AK (mg kg^−1^)
Faba bean	38	7.3 ± 0.77aA	2.31 ± 1.33aA	99.32 ± 56.72aA	51.02 ± 38.64aA	154.68 ± 57.35bA
Pea	26	7.5 ± 0.73aA	1.91 ± 0.79bA	85.21 ± 48.31aA	58.07 ± 79.77aA	147.62 ± 71.90bA
Adzuki bean	17	7.6 ± 0.61aA	1.94 ± 1.39aA	84.27 ± 39.42aA	51.71 ± 47.42aA	141.20 ± 79.55bA
Mung bean	48	7.1 ± 1.06bA	1.82 ± 1.18bA	81.74 ± 32.62aA	43.76 ± 47.95aA	144.00 ± 60.26bB
Common bean	21	7.7 ± 0.74aA	2.89 ± 2.27aA	101.65 ± 60.20aA	50.47 ± 55.36aA	212.27 ± 123.04aA

The different capital and lowercase letters mean significantly different at 0.01 and 0.05 probability levels, respectively.

**Table 8:** The soil nutrients content of sites for production of different edible type faba bean and pea.

Edible type	Distribution	Soil pH	SOM (%)	AN (mg kg^−1^)	AP (mg kg^−1^)	AK (mg kg^−1^)
Vegetable	Eastern and Southwestern China	7.2 ± 0.76bB	2.05 ± 1.19aA	92.14 ± 58.73aA	56.59 ± 61.50aA	142.42 ± 57.32bA
Grain	Northwestern China	7.9 ± 0.43aA	2.55 ± 0.89aA	99.26 ± 24.93aA	43.27 ± 44.84aA	188.65 ± 73.76aA

The different capital and lowercase letters mean significantly different at 0.01 and 0.05 probability levels, respectively.

**Table 9:** Classification of soil PH.

PH	<5.5	5.5~6.5	6.5~7.5	7.5~8.5	>8.5
Response level	Strongly acid	Slightly acid	Neutral	Slightly alkaline	Strongly alkaline

**Table 10:** Classification of soil nutrients.

Classes	SOM(%)	AN (mg kg^−1^)	AP (mg kg^−1^)	AK (mg kg^−1^)
1	>4	>150	>40	>200
2	3~4	120~150	20~40	150~200
3	2~3	90~120	10~20	100~150
4	1~2	60~90	5~10	50~100
5	0.6~1	30~60	3~5	30~50
6	<0.6	<30	<3	<30
