# Supplementary material for: Soil Fertility Map for Food Legumes Production Areas in China
Source: Sci Rep. 2016 May 23;6:26102. doi: 10.1038/srep26102 (PMC4876514; doi:10.1038/srep26102)
Supplement: Supplementary Information [file srep26102-s1.pdf]

Supplementary Information for

Soil Fertility Map for Food Legumes Production Areas in China

L. Li, T. Yang, R. Redden, W. He, X.X. Zong

Table S1. The soil nutrients content of food legumes production areas in China

No.	Cluster	Province	Location	Longitude	Latitude	Soil pH	SOM（%）	AN （mg kg^{-1}）	AP (mg kg^{-1})	AK (mg kg^{-1}）
					**Faba bean**					
1	1	Anhui	Lixin	116.34	33.11	6.9	2.0	115.0	92.2	160.3
2	1	Anhui	Luyang	117.14	31.54	7.5	1.7	82.0	39.9	110.0
3	1	Gansu	Hezheng	103.22	35.35	7.4	3.8	148.2	19.3	267.0
4	1	Gansu	Jishishan	102.89	35.68	7.6	1.0	87.0	20.6	187.5
5	1	Gansu	Kangle	103.55	35.29	7.1	2.7	138.5	19.5	167.6
6	1	Gansu	Linxia	102.96	35.52	8.0	2.9	112.7	17.0	122.8
7	1	Gansu	Weiyuan	104.06	35.03	7.2	1.9	80.5	28.4	115.9
8	1	Hubei	Wuhan	114.31	30.49	7.6	1.8	56.0	67.1	196.3
9	1	Hubei	Gucheng	111.65	32.39	6.0	1.0	70.0	13.2	124.9
10	1	Hubei	Jingzhou	112.34	30.28	7.6	1.1	105.0	71.4	78.3
11	1	Hubei	Badong	110.31	31.04	6.4	1.3	59.5	83.7	124.9
12	1	Jiangsu	Rudong	121.30	32.25	8.0	1.8	93.0	153.3	166.0
13	1	Sichuan	Nanchong	105.91	30.73	7.2	3.6	96.6	102.7	167.6
14	1	Sichuan	Neijiang	105.05	29.58	7.1	1.2	67.6	99.7	104.0
15	1	Sichuan	Pingwu	104.54	30.35	7.6	2.6	102.4	16.1	200.0
16	1	Sichuan	Jianyang	104.54	30.41	6.8	1.4	92.1	50.6	139.7
17	1	Xinjiang	Wulumuqi	88.25	43.70	8.1	3.0	103.0	43.1	146.0
18	1	Yunnan	Songming	103.11	25.35	7.1	1.5	155.0	76.2	186.4
19	1	Yunnan	Midu	100.52	25.38	7.4	1.4	101.0	72.1	102.0
20	1	Yunnan	Luliang	103.75	25.04	7.8	4.7	105.0	27.8	179.5
21	1	Yunnan	Qilin	103.85	25.48	7.7	3.5	77.0	22.3	204.8
22	1	Yunnan	Sizong	103.80	24.76	7.8	5.0	112.0	28.0	167.4
23	1	Yunnan	Eryuan	100.19	25.69	6.2	3.1	266.0	98.5	250.0
24	1	Yunnan	Xianyun	100.54	25.47	7.0	2.8	137.0	88.6	301.1
25	1	Hebei	Chongli	115.45	41.62	7.8	2.3	56.0	113.5	157.8
26	1	Hebei	Zhangbei	114.70	41.15	8.2	2.1	45.5	50.0	157.8
27	1	Hebei	Zhangbei	115.35	41.32	7.7	2.2	59.5	43.2	157.8
28	1	Chongqing	Hechuan	106.46	30.24	7.3	1.5	38.5	32.7	221.6
29	1	Chongqing	Yongchuan	105.84	29.18	5.2	1.7	101.5	5.3	201.2
30	1	Qinghai	Xining	101.74	36.56	7.2	1.9	93.0	47.0	150.0

31	1	Hubei	Dongxihu	114.08	30.72	7.0	1.5	56.0	106.0	240.9
32	2	Anhui	Wuhe	117.77	33.06	8.3	1.4	115.0	3.3	85.9
33	2	Jiangsu	Tongzhou	121.25	32.16	8.0	1.3	66.0	15.5	118.0
34	2	Hebei	Guyuan	116.17	42.12	8.2	0.6	28.0	14.5	115.6
35	2	Chongqing	Zhongxian	107.80	30.35	7.8	1.4	49.0	7.7	126.5
36	3	Yunnan	Zhanyi	103.84	25.61	7.8	6.1	101.5	23.2	75.9
37	3	Yunnan	Dali	100.43	25.50	7.9	5.7	331.0	12.7	60.4
38	4	Sichuan	Dazhu	107.12	30.42	4.9	1.3	71.7	112.8	38.5
					**Pea**					
1	1	Anhui	Hefei	117.26	31.89	6.4	1.5	73.5	11.3	105.0
2	1	Hubei	Gucheng	111.56	32.27	6.7	0.9	42.0	20.9	139.7
3	1	Sichuan	Jinjiang	104.11	30.61	7.1	1.1	106.3	23.1	79.3
4	1	Yunnan	Dali	100.07	25.78	7.0	1.8	253.0	76.1	59.7
5	1	Jiangsu	Rugao	120.77	32.20	7.5	2.2	119.0	45.6	90.0
6	1	Shandong	Laiyang	120.99	37.16	6.1	1.2	63.0	96.3	139.7
7	1	Shandong	Lichang	120.42	36.15	6.6	1.7	81.0	31.3	184.9
8	1	Shandong	Lichang	120.39	36.20	6.3	1.7	42.0	273.4	171.1
9	1	Sichuan	Xindu	104.21	30.78	7.1	2.1	83.7	99.6	75.4
10	1	Sichuan	Lezhi	105.02	30.42	7.1	3.1	54.8	29.0	92.1
11	2	Liaoning	Dengta	123.43	41.43	6.2	2.0	193.0	310.5	246.8
12	3	Yunnan	Jiangchuan	102.66	24.28	7.1	3.1	99.9	27.1	223.3
13	3	Gansu	Anding	104.59	35.56	8.3	2.1	101.0	112.6	333.7
14	3	Gansu	Lintao	103.77	35.16	8.0	2.8	119.0	166.7	277.1
15	3	Shanxi	Dingxiang	112.82	38.64	7.8	4.3	78.5	15.1	139.7
16	3	Xinjiang	Mulei	90.27	43.75	8.1	2.5	99.0	30.7	259.0
17	4	Hubei	Wuhan	114.24	30.47	7.8	1.2	49.0	4.9	100.0
18	4	Chongqing	Tongnan	105.87	30.34	7.8	1.0	21.0	1.4	126.5
19	4	Chongqing	Wushan	109.81	31.07	8.1	1.7	42.0	4.3	157.8
20	4	Anhui	Xiaoxian	116.79	34.07	7.7	0.8	59.5	14.1	101.0
21	4	Gansu	Longxi	104.55	35.31	8.3	1.9	69.0	33.5	100.0
22	4	Gansu	Huining	105.00	35.48	8.3	1.6	70.0	27.3	202.4
23	4	Gansu	Tongwei	105.17	35.40	8.3	2.6	84.0	28.7	133.7
24	4	Jiangsu	Haimen	121.39	31.25	8.0	1.6	71.0	12.2	86.0
25	4	Jiangsu	Qidong	121.65	31.84	8.0	1.6	90.0	12.2	154.0
26	4	Shandong	Binzhou	117.95	37.43	8.1	1.6	51.2	1.9	60.2
					**Adzuki bean**					
1	1	Heilongjiang	Gannan	123.57	47.72	6.0	5.4	215.0	19.6	244.5
2	2	Heilongjiang	Longjiang	123.20	47.34	7.7	3.8	112.6	14.8	250.6
3	2	Hebei	Xuanhua	114.83	40.68	7.5	4.7	115.5	53.7	234.9
4	2	Hebei	Xiongxian	116.24	39.00	8.4	1.8	75.0	8.7	257.0
5	2	Hebei	Gaoyang	115.86	38.74	8.3	1.3	88.0	47.1	227.0
6	3	Liaoning	Kangping	123.32	42.95	8.1	1.2	49.0	12.3	58.4
7	3	Liaoning	Lingyuan	119.41	43.10	7.5	1.2	61.6	21.1	70.5
8	3	Liaoning	Zhangwu	122.36	42.40	6.6	0.8	47.0	14.5	62.7

9	3	Hebei	Qiutou	114.48	38.07	7.2	2.1	103.0	12.4	96.9
10	3	Hebei	Yixian	115.51	39.45	8.5	0.6	53.0	19.8	116.0
11	3	Beijing	Changping	116.30	40.09	7.8	1.2	69.3	32.1	82.2
12	3	Beijing	Fangshan	116.11	39.69	7.7	0.7	53.9	21.6	64.4
13	4	Hebei	Leting	118.80	39.43	7.6	1.5	80.5	116.6	187.5
14	4	Hebei	Qianan	118.81	40.09	7.6	1.7	83.7	125.3	80.1
15	4	Hebei	Qianxi	118.36	40.20	7.6	2.4	74.1	103.2	40.4
16	4	Hebei	Yutian	117.66	39.64	7.6	1.4	74.1	124.1	195.5
17	4	Hebei	Zunhua	117.96	40.89	7.5	1.2	77.3	132.1	131.8
					**Mung bean**					
1	1	Shandong	Jiaonan	119.92	35.85	7.5	2.5	70.9	127.0	139.8
2	1	Anhui	Woyang	116.04	33.61	7.7	1.9	117.0	85.5	170.3
3	1	Anhui	Mingguang	118.08	32.78	6.3	1.2	84.0	11.0	156.8
4	1	Henan	Fangcheng	112.89	33.15	6.1	1.0	72.0	21.0	174.4
5	1	Henan	Yangji	113.01	33.29	6.5	1.2	76.0	109.8	156.3
6	1	Henan	Sheqi	113.05	32.82	5.6	1.7	87.0	38.5	138.2
7	1	Henan	Taihe	112.94	32.86	4.7	1.2	91.0	69.7	158.3
8	1	Henan	Shangtun	112.75	32.57	6.1	1.5	65.0	32.6	232.7
9	1	Henan	Tanghe	112.74	32.80	6.0	1.3	75.0	12.8	148.2
10	1	Henan	Dengzhou	111.99	32.70	6.5	1.9	115.0	72.5	256.8
11	1	Henan	Xinye	112.48	32.49	6.4	1.9	107.0	49.6	206.5
12	1	Henan	Wancheng	112.57	32.94	6.0	1.2	94.0	39.1	216.6
13	1	Heilongjiang	Dumeng	124.35	46.29	6.0	1.0	88.7	19.3	106.0
14	1	Heilongjiang	Meilisi	123.74	47.35	8.0	1.6	119.4	75.2	202.4
15	1	Heilongjiang	Tailai	123.44	46.46	5.8	1.1	81.9	91.3	108.4
16	1	Jilin	Baicheng	122.65	45.72	7.1	3.3	103.1	15.7	139.8
17	1	Jilin	Baichengyuan	122.78	45.60	7.1	1.3	112.7	57.7	247.1
18	1	Jilin	Daan	123.30	45.58	7.0	1.5	74.1	12.0	179.6
19	1	Jilin	Taoer	122.80	45.43	7.1	3.2	190.0	12.6	139.8
20	1	Jilin	Xinxiang	122.83	44.70	7.1	1.5	51.5	17.3	183.5
21	1	Jilin	Zhenlai	123.13	45.93	7.0	2.5	116.0	22.2	104.0
22	1	Jilin	Zhennan	122.97	45.78	7.2	3.1	112.7	17.3	171.6
23	1	Liaoning	Kazuo	119.75	41.24	8.7	1.6	58.0	71.5	103.8
24	1	Inner Mongolia	Tuquan	121.88	45.62	7.8	2.9	180.9	25.8	172.3
25	1	Shandong	Changyi	119.39	36.85	7.5	1.4	109.5	153.3	294.9
26	1	Shandong	Kuiju	119.40	36.83	7.0	1.2	42.0	229.1	253.0
27	1	Shandong	Zangnan	119.92	35.86	6.6	4.9	51.2	42.1	136.1
28	1	Shanxi	Guayuan	113.86	40.09	7.9	1.0	78.5	14.0	180.7
29	1	Hebei	Qingyuan	115.42	38.73	7.5	0.8	98.0	22.4	126.0
30	1	Hebei	Lixian	115.63	38.42	8.2	1.6	90.0	60.2	126.0
31	1	Hebei	Baoding	115.45	38.83	8.0	1.6	60.0	25.0	130.0
32	2	Beijing	Mentougou	116.10	40.00	7.5	1.2	57.8	31.7	71.6
33	2	Beijing	Miyun	117.13	40.54	7.3	1.2	61.6	23.0	79.7
34	2	Beijing	Beijing	116.30	40.09	7.7	1.0	53.9	20.5	73.9

35	2	Beijing	Yanqing	116.23	40.43	6.6	1.1	34.7	22.8	57.4
36	2	Hubei	Badong	110.31	31.06	6.7	1.9	38.5	8.5	173.5
37	2	Jilin	Qianguo	124.84	45.10	8.4	1.9	86.0	12.3	77.9
38	2	Jilin	Taonan	122.81	45.33	8.7	1.3	54.0	7.3	76.8
39	2	Jilin	Tongyu	124.37	44.63	8.7	1.6	89.0	9.8	97.8
40	2	Jilin	Changling	123.73	44.21	8.8	2.1	82.0	10.4	107.8
41	2	Jilin	Zhenlai	122.89	45.56	9.2	2.2	98.0	11.8	102.6
42	2	Liaoning	Fumeng	121.94	41.99	7.5	1.3	53.9	33.7	60.0
43	2	Inner Mongolia	Chifeng	120.19	44.26	7.9	0.8	41.0	4.7	100.0
44	2	Inner Mongolia	Shaihan	111.84	40.83	7.7	1.7	58.0	5.3	87.9
45	2	Hebei	Yangyuan	114.28	40.20	8.2	0.7	28.0	15.4	261.4
46	3	Sichuan	Dazhou	107.49	31.23	5.0	1.9	92.1	181.5	79.5
47	3	Shandong	Huangdao	119.75	35.77	4.8	2.2	71.7	42.1	60.2
48	4	Shanxi	Datong	114.34	40.45	7.8	7.8	51.2	6.4	84.3
					**Common bean**					
1	1	Yunnan	Fuyuan	104.26	25.51	6.8	6.5	105.0	29.6	396.3
2	1	Guizhou	Weining	104.39	27.10	7.6	6.2	189.0	133.8	285.5
3	1	Guizhou	Dafang	105.73	27.22	8.0	7.9	220.0	69.4	137.3
4	1	Guizhou	Nayong	105.48	26.97	7.9	4.1	161.0	57.7	416.8
5	2	Guizhou	Zhijin	105.87	26.77	7.7	3.5	156.0	14.3	124.1
6	2	Inner Mongolia	Fengzhen	112.88	40.65	7.5	2.1	71.7	26.1	172.3
7	2	Inner Mongolia	Liangcheng	112.31	40.81	7.8	1.2	71.7	8.1	132.5
8	2	Shanxi	Taiyuan	112.43	37.41	8.4	3.6	46.2	20.0	88.9
9	2	Shanxi	Hequ	111.35	39.24	7.9	1.2	37.5	10.9	115.6
10	2	Shanxi	Kelan	111.61	38.77	7.8	1.2	54.6	9.3	160.2
11	2	Shanxi	Yuci	112.67	37.55	8.1	1.4	51.2	12.7	139.7
12	2	Shanxi	Tianzhen	112.46	40.24	7.7	1.5	85.3	23.9	124.1
13	2	Shanxi	Youyu	113.62	40.13	7.7	0.9	58.0	6.8	120.5
14	2	Xinjiang	Aletai	88.25	47.70	7.9	2.1	114.0	37.5	235.0
15	2	Xinjiang	Buerjin	86.98	47.85	8.4	1.0	66.0	49.0	102.0
16	2	Hebei	Kangbao	114.48	42.13	8.1	1.6	52.5	5.7	78.3
17	3	Shandong	Pingdu	119.96	36.71	7.2	2.1	88.7	140.5	208.4
18	3	Shandong	Lingou	118.69	36.55	7.3	1.9	90.2	159.5	275.0
19	3	Shanxi	Yuxian	113.05	38.14	7.8	1.7	92.1	28.2	486.7
20	3	Xinjiang	Fuyun	88.72	46.38	8.2	1.3	70.0	30.3	414.0
21	4	Guizhou	Hezhang	105.07	27.41	4.9	7.7	254.0	186.5	244.5

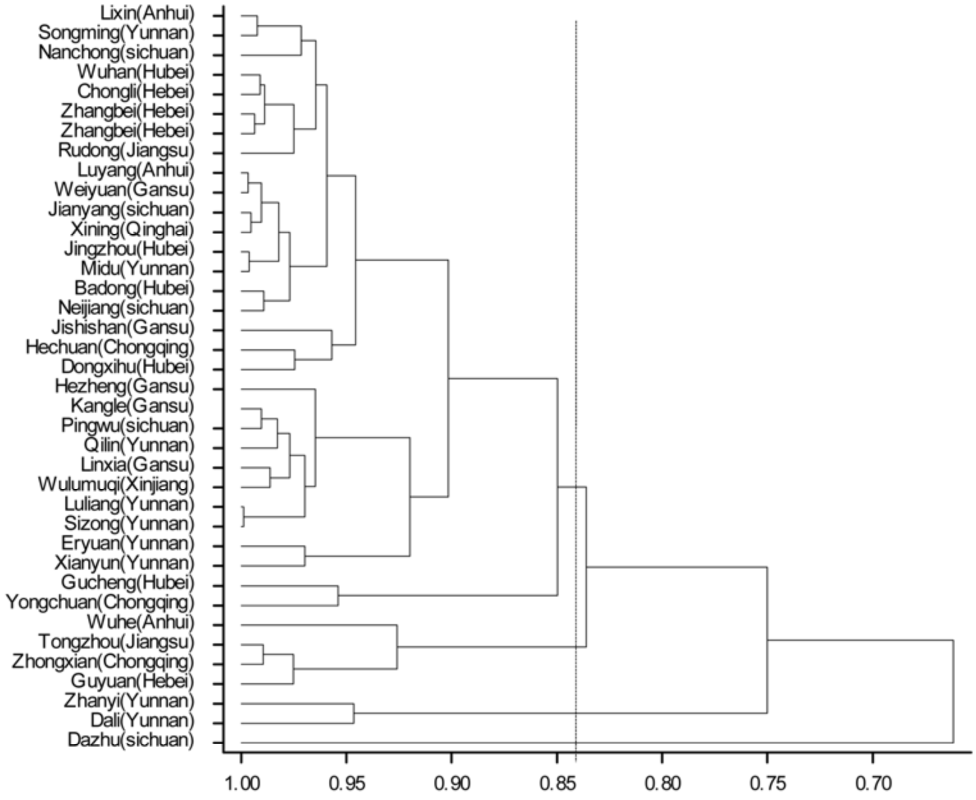

Faba bean

Figure S1. Cluster tree of 38 soil samples of the faba bean production areas.

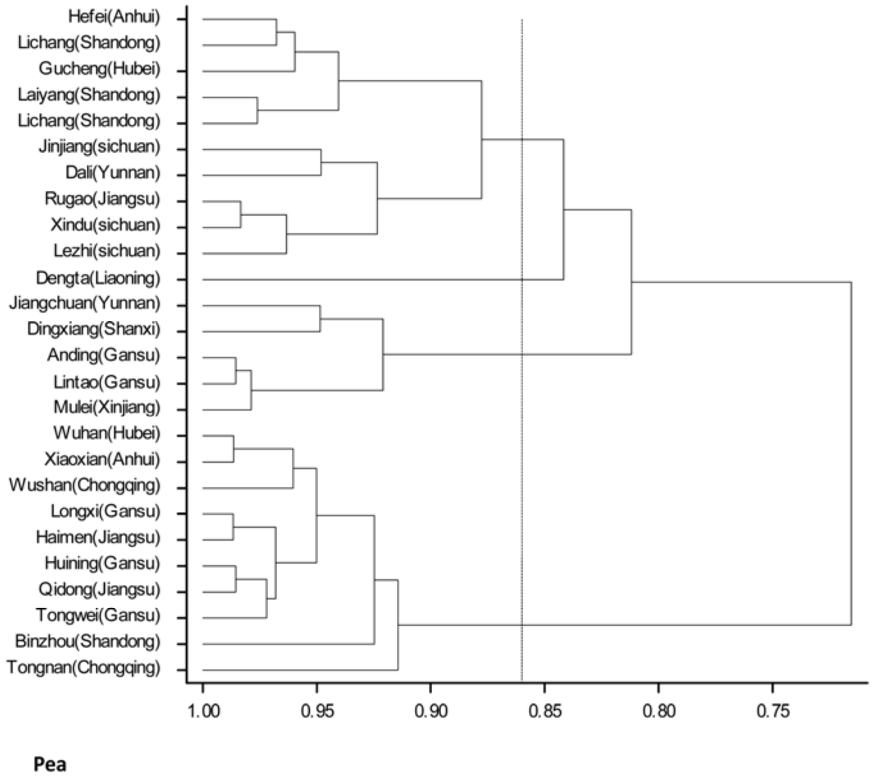

Figure S2. Cluster tree of 26 soil samples of the pea production areas.

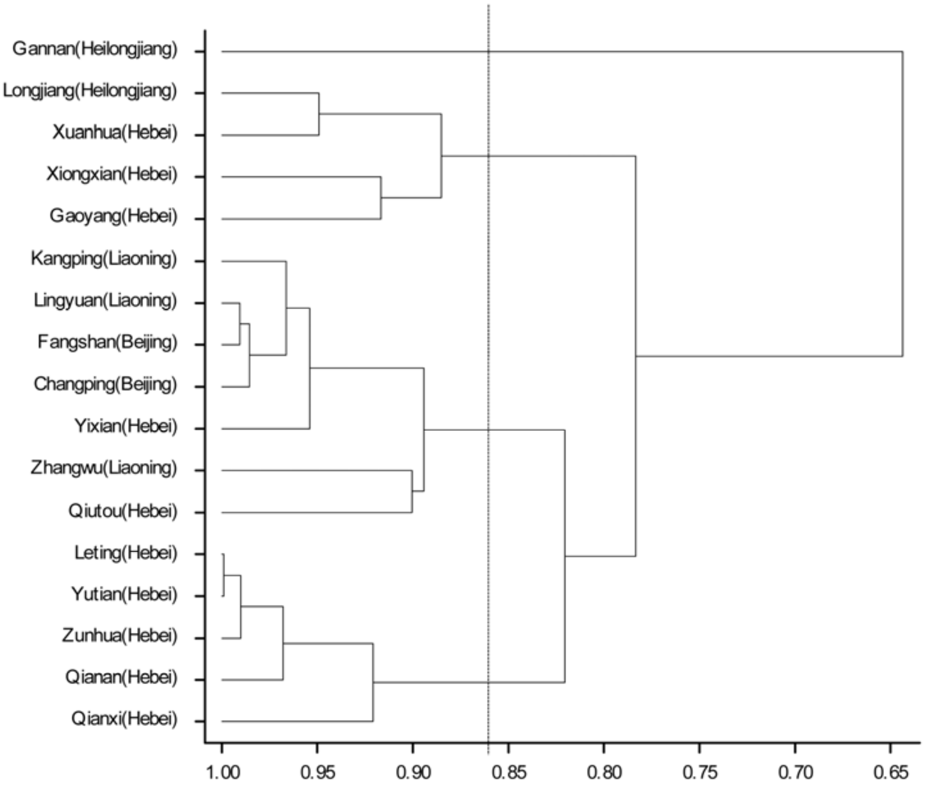

Adzuki bean

Figure S3. Cluster tree of 17 soil samples of the adzuki bean production areas.

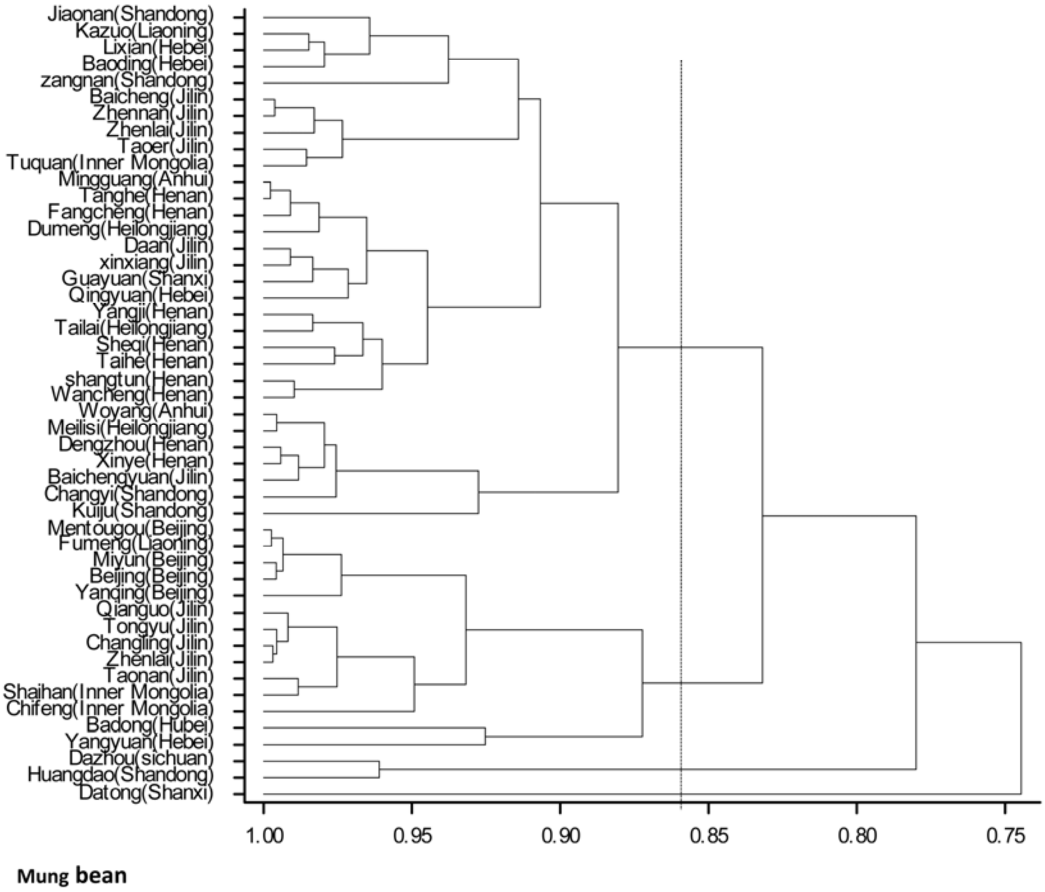

Figure S4. Cluster tree of 48 soil samples of the mung bean production areas.

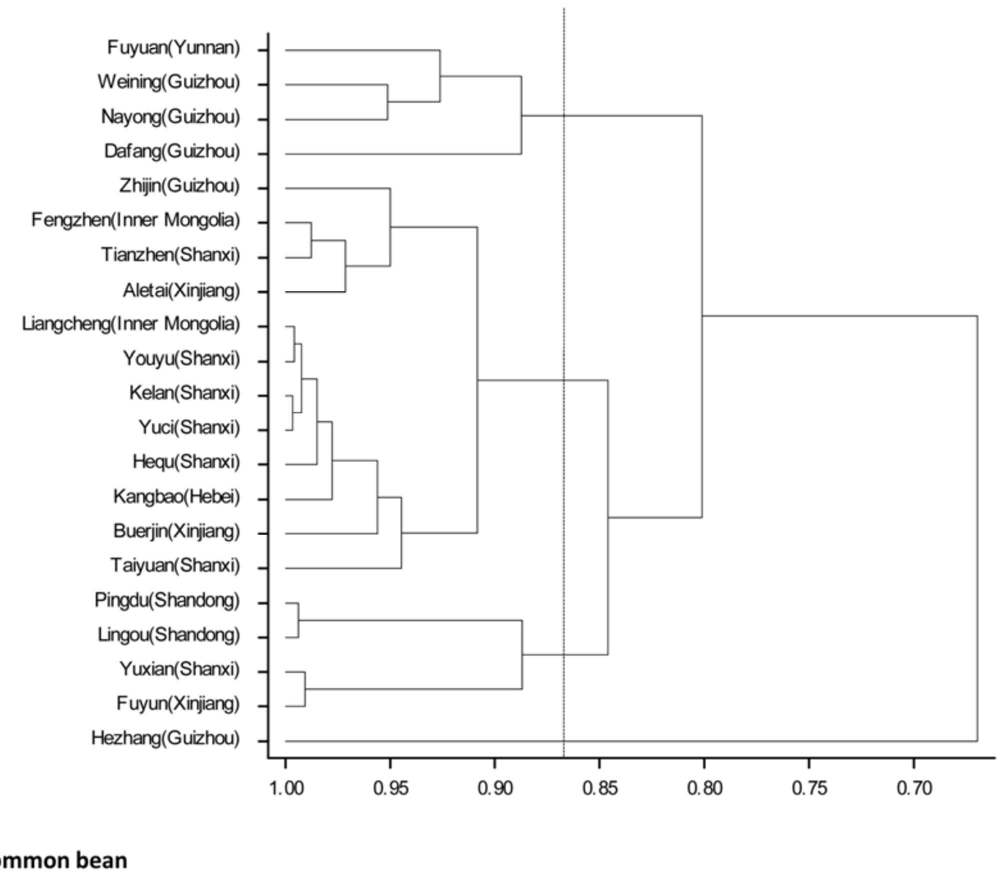

Figure S5. Cluster tree of 21 soil samples of the common bean production areas.
